# Successful Mediastinal Cryobiopsy Using the Slim UCP190F EBUS Bronchoscope and a 25G Needle: Demonstrating the Efficacy of the Ariza‐Pallares Approach

**DOI:** 10.1002/rcr2.70561

**Published:** 2026-04-20

**Authors:** Sammy Onyancha, Gernot Rohde

**Affiliations:** ^1^ Department of Pulmonology St. Elisabethen Krankenhaus Frankfurt Germany; ^2^ Department of Respiratory Medicine Universitätsklinikum Marburg Marburg Germany

**Keywords:** Ariza‐Pallares, CryoEBUS, EBUS, mediastinal Cryobiopsy

## Abstract

Mediastinal cryobiopsy is emerging as a powerful adjunct to traditional EBUS‐TBNA, offering larger, architecturally preserved tissue samples. We describe a case demonstrating successful mediastinal cryobiopsy using the newly developed UCP190F endobronchial ultrasound (EBUS) bronchoscope in a patient with a right upper lobe lesion and mediastinal lymphadenopathy. Owing to the device's narrow 1.7 mm working channel, only 25‐gauge needles (0.4 mm diameter) can be used, raising concerns regarding the feasibility of creating a sufficiently stable access tract for introducing a 1.1 mm cryoprobe for mediastinal cryobiopsy. Using the Ariza‐Pallares four‐step method, a navigable tunnel into station 7 was successfully created, enabling safe cryobiopsy and yielding high‐quality histologic samples. This case demonstrates that mediastinal cryobiopsy can be successfully performed even with very small‐gauge needles and with new‐generation thin convex‐probe scopes, expanding the applicability of cryobiopsy across EBUS platforms.

## Graphical abstract

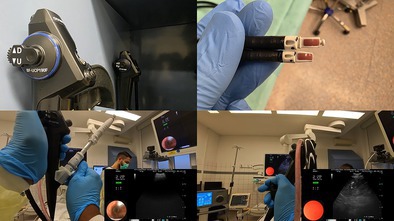

## 1 Introduction

Mediastinal cryobiopsy is emerging as a powerful adjunct to traditional EBUS‐TBNA, offering larger, architecturally preserved tissue samples with superior diagnostic yield for lymphoproliferative diseases, granulomatous disorders and advanced lung cancer molecular profiling [1].

The Ariza‐Pallares method provides a standardised four‐step ultrasound‐guided approach to mediastinal cryobiopsy—planning, puncture, tunnelling and cryobiopsy—which removes the need for a high‐frequency needle knife and allows safe access even in challenging stations [2].

The new BF‐UCP190F EBUS bronchoscope extends the reach of convex‐probe EBUS into segmental and subsegmental bronchi due to its slim 5.9 mm distal end and expanded 170° upward angulation, but its 1.7 mm working channel restricts device compatibility to a 25‐gauge TBNA needle. Whether such a narrow needle can reliably create a tract adequate for a 1.1 mm cryoprobe has not been previously documented. This case illustrates the feasibility of this approach.

## 2 Case Report

**FIGURE 1:**
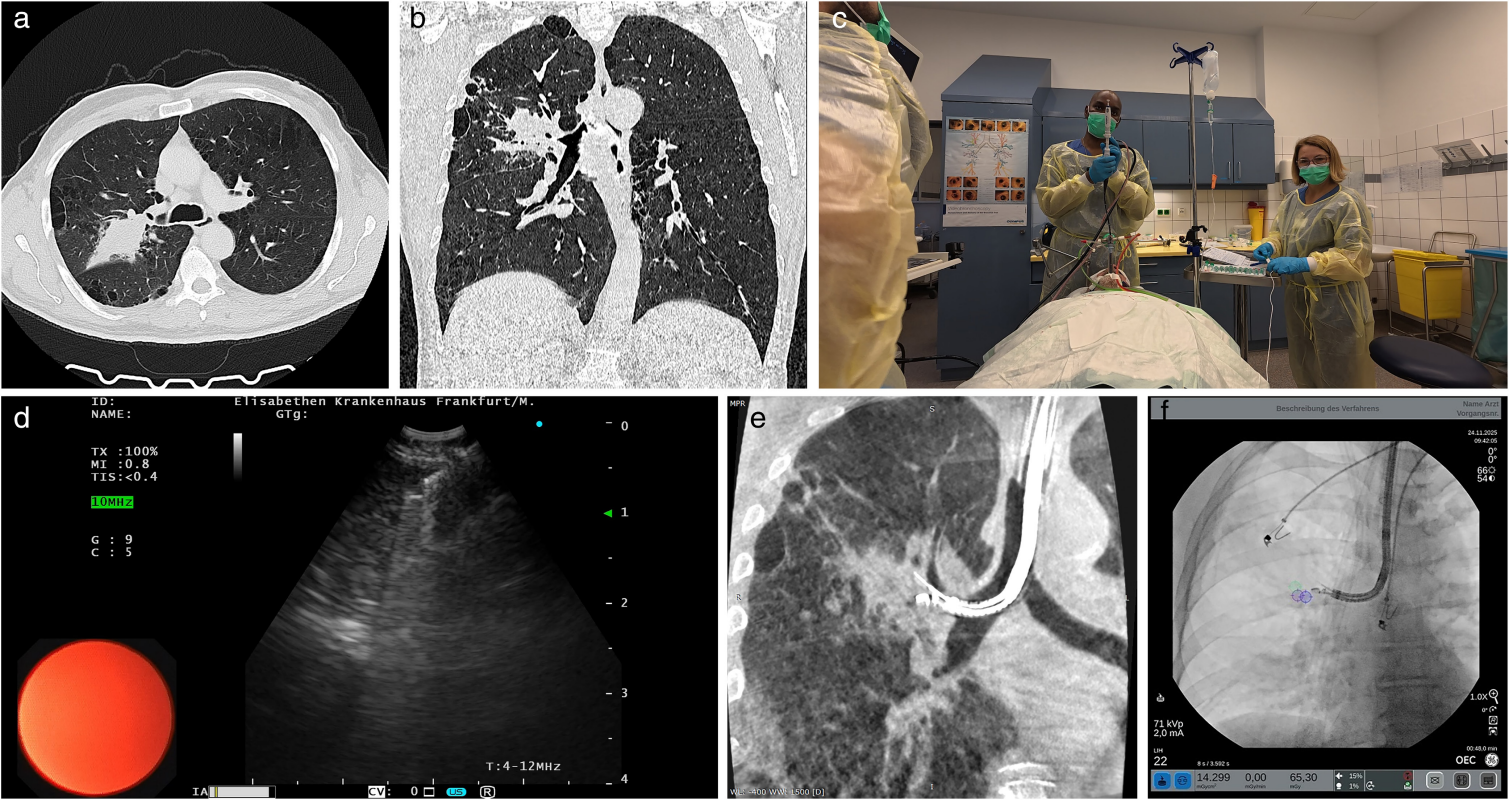
(a) Axial CT‐Scan showing a lesion in the right upper lobe. (b) Coronal CT‐Scan showing a lesion in the right upper lobe. (c) EBUS‐TBNA being carried out with 25G needle. (d) EBUS image of cryoprobe in the Lymph node. (e) CBCT‐Image of EBUS‐Bronchoscope in the right upper lobe. (f) Augmented fluoroscopy after CBCT showing TBNA needle in the upper lobe lesion.

A 51‐year‐old former smoker was referred for evaluation by dyspnoea and night sweats. Computer tomography revealed an upper lobe pulmonary lesion with associated mediastinal lymphadenopathy (Figure 1a,b).

Flexible bronchoscopy was performed under conscious sedation using the new BF‐UCP190F EBUS bronchoscope (Olympus Medical Systems, Japan), chosen for its extended reach and slim 5.9 mm distal end, which facilitated navigation into the upper lobe bronchial segments. Because the device's 1.7 mm working channel only accommodates a 25‐gauge TBNA needle, sampling was performed using a ViziShot 2 25G needle (0.4 mm diameter).

Following the Ariza‐Pallares methodology [2], an ultrasound‐guided four‐step process was used. Lymph node station 7 was identified as the optimal target. Doppler imaging confirmed safe vascular patterns with a clear bronchial‐nodal interface suitable for TBNA access.

Two TBNA passes were performed into the node through the thinnest mucosal region, avoiding cartilage and vascular structures (Figure 1c). Despite the very small diameter of the 25G needle, a visible TBNA tract was created under EBUS visualisation.

Using the same needle trajectory, additional non‐aspiration passes were performed to widen and stabilise the tract. Ultrasound imaging revealed the characteristic needle‐trace line and disruption of the lymph‐node capsule, indicating successful tunnel formation.

A 1.1 mm cryoprobe was advanced through the working channel using real‐time ultrasound guidance (Figure 1d). The probe entered the needle tract and was positioned centrally within the node. Three cryobiopsies were obtained with a 4 s freeze time, and each biopsy demonstrated excellent tissue volume and architecture (Video 1).

**VIDEO 1:** Step by step—mediastinal cryobiopsy with 25G needle. Video content can be viewed at https://onlinelibrary.wiley.com/doi/10.1002/rcr2.70561.

Following the cryobiopsy, the EBUS bronchoscope was inserted into the posterior segment of the right upper lobe, and the suspected lesion was biopsied using the TBNA needle under sonographic and fluoroscopic guidance (Figure 1e,f). No bleeding or airway compromise occurred. A postinterventional chest x‐ray ruled out a pneumothorax. The total procedural duration was 37 min.

The patient recovered uneventfully and was discharged after an overnight observation. Histopathology confirmed metastatic non‐small cell lung carcinoma with adequate material for complete molecular analysis.

## 3 Discussion

This case demonstrates, for the first time to our knowledge, the feasibility of performing mediastinal cryobiopsy using a 25‐gauge needle to create the access tract. Prior studies describe the use of significantly larger needles (22G, 21G and even 19G) to generate a canal sufficiently wide for a 1.1 mm cryoprobe [3, 4, 5].

Concern has existed that the 0.4 mm diameter of a 25G needle would be insufficient for the passage of a cryoprobe without requiring a needle‐knife incision. However, this case shows that the Ariza‐Pallares approach, especially the ultrasound‐guided tunnelling technique, allows reliable access regardless of needle gauge.

The BF‐UCP190F bronchoscope is designed for deeper peripheral access and features a slim distal profile and enhanced upward angulation, but the trade‐off is a narrow 1.7 mm instrument channel, restricting it to 25G TBNA needles.

This limitation could have precluded cryobiopsy, but the successful cryoprobe introduction in this case supports the broader applicability of mediastinal cryobiopsy on thin‐convex EBUS platforms.

The ability to obtain high‐quality cryobiopsy samples through this device may expand the diagnostic capabilities of the UCP190F, particularly valuable when larger tissue samples are needed for molecular characterisation, immunohistochemistry or the diagnosis of lymphoproliferative disease.

However, this report represents a single‐case experience, and the conclusions regarding feasibility should therefore be interpreted with caution. While the successful creation of a cryobiopsy tract using a 25G needle suggests that mediastinal cryobiopsy may be technically achievable even with ultra‐thin convex‐probe EBUS platforms, broader validation in larger patient cohorts will be necessary. Prospective studies evaluating procedural success, safety and diagnostic yield across different operators and clinical settings will be important to confirm the generalisability of this approach and to define its role in routine clinical practice.

## Author Contributions

All the authors contributed to the manuscript. The first draft was written by Sammy Onyancha, and all authors commented on previous versions of the manuscript. All authors have read and approved the final manuscript.

## Funding

The authors have nothing to report.

## Consent

The authors declare that written informed consent was obtained for the publication of this manuscript and accompanying images using the form provided by the Journal.

## Conflicts of Interest

The authors declare no conflicts of interest.

## Data Availability

The data that support the findings of this study are available on request from the corresponding author. The data are not publicly available due to privacy or ethical restrictions.
